# The Lectin Pathway of Complement Activation Is a Critical Component of the Innate Immune Response to Pneumococcal Infection

**DOI:** 10.1371/journal.ppat.1002793

**Published:** 2012-07-05

**Authors:** Youssif M. Ali, Nicholas J. Lynch, Kashif S. Haleem, Teizo Fujita, Yuichi Endo, Soren Hansen, Uffe Holmskov, Kazue Takahashi, Gregory L. Stahl, Thomas Dudler, Umakhanth V. Girija, Russell Wallis, Aras Kadioglu, Cordula M. Stover, Peter W. Andrew, Wilhelm J. Schwaeble

**Affiliations:** 1 Department of Infection, Immunity and Inflammation, University of Leicester, Leicester, United Kingdom; 2 Faculty of Pharmacy, University of Mansoura, Mansoura, Egypt; 3 Department of Immunology, Fukushima Medical University, Fukushima, Japan; 4 Institute of Molecular Medicine, University of Southern Denmark, Odense, Denmark; 5 Department of Anesthesiology, Perioperative and Pain Medicine, Harvard Medical School, Boston, Massachusetts, United States of America; 6 Omeros Corporation, Seattle, Washington, United States of America; The University of Texas Health Science Center at San Antonio, United States of America

## Abstract

The complement system plays a key role in host defense against pneumococcal infection. Three different pathways, the classical, alternative and lectin pathways, mediate complement activation. While there is limited information available on the roles of the classical and the alternative activation pathways of complement in fighting streptococcal infection, little is known about the role of the lectin pathway, mainly due to the lack of appropriate experimental models of lectin pathway deficiency. We have recently established a mouse strain deficient of the lectin pathway effector enzyme mannan-binding lectin associated serine protease-2 (MASP-2) and shown that this mouse strain is unable to form the lectin pathway specific C3 and C5 convertases. Here we report that MASP-2 deficient mice (which can still activate complement via the classical pathway and the alternative pathway) are highly susceptible to pneumococcal infection and fail to opsonize *Streptococcus pneumoniae* in the none-immune host. This defect in complement opsonisation severely compromises pathogen clearance in the lectin pathway deficient host. Using sera from mice and humans with defined complement deficiencies, we demonstrate that mouse ficolin A, human L-ficolin, and collectin 11 in both species, but not mannan-binding lectin (MBL), are the pattern recognition molecules that drive lectin pathway activation on the surface of *S. pneumoniae*. We further show that pneumococcal opsonisation via the lectin pathway can proceed in the absence of C4. This study corroborates the essential function of MASP-2 in the lectin pathway and highlights the importance of MBL-independent lectin pathway activation in the host defense against pneumococci.

## Author Summary

*Streptococcus pneumoniae* is a major human pathogen that causes pneumonia, septicemia and meningitis. The host defense against pneumococci is largely dependent on complement, a system of blood proteins which, when activated, attach to bacteria, targeting them for clearance by phagocytes. There are three routes of complement activation: The classical, lectin and alternative pathways. Limited information is available on the roles of the classical and alternative pathways in fighting pneumococci; the role of the lectin pathway has escaped the attention of previous research. This work demonstrates that the lectin pathway is critical in fighting pneumococcal infection. Of the five different lectin pathway recognition molecules in human serum, only L-ficolin and collectin 11 activate complement on pneumococci. Human mannose-binding lectin (MBL), the best-known lectin pathway pattern recognition molecule, has no role whatsoever in fighting pneumococci. Similarly, in mouse serum, only ficolin A and collectin 11 drive complement activation on *S. pneumoniae*. Hence, MBL deficient mice are not compromised in pneumococcal infection, while ficolin A deficient mice and mice deficient of the key lectin pathway enzyme MBL-associated serine protease-2 (MASP-2) are exquisitely susceptible to infection. This work explains why MBL deficiency, the most frequent hereditary immune deficiency, does not predispose to pneumococcal disease.

## Introduction

*Streptococcus pneumoniae* infection is a major cause of pneumonia, otitis media, septicemia and meningitis [1], [2]. Complement–driven opsonophagocytosis is a prominent feature of the host response to pneumococcal infections, [3].

Complement provides protection against invading microorganisms through both antibody-dependent and -independent mechanisms. It mediates many cellular and humoral interactions within the immune response, including chemotaxis, phagocytosis, cell adhesion, and B-cell differentiation. Three different pathways initiate the complement cascade, which are known as the classical, alternative and lectin pathways.

In the classical pathway, the recognition subcomponent C1q binds to a variety of targets - most prominently immune complexes - to initiate the step-wise activation of associated serine proteases, C1r and C1s. Activated C1s cleaves C4 into C4a and C4b and then cleaves C4b-bound C2 to generate the C3 convertase, C4b2a, which converts the abundant plasma protein C3 into C3a and C3b; C3b is the major opsonin of the complement system. Accumulation of C3b in close proximity to the C4b2a complex leads to the formation of the C5 convertase, C4b2a(C3b)_n_, which initiates the terminal pathway of complement activation.

In the alternative pathway, spontaneous low-level hydrolysis of C3 leads to deposition of C3b on cell surfaces, triggering complement activation on foreign cells. Host cells are protected by surface regulatory proteins that suppress complement activation.

Like the alternative pathway, the lectin pathway may be activated in the absence of immune complexes. Activation is initiated by the binding of a multimolecular lectin pathway activation complex to pathogen-associated molecular patterns (PAMPs), mainly carbohydrate structures present on microorganisms or aberrant glycocalyx patterns on apoptotic, necrotic, malignant or oxygen-deprived cells [4], [5]. Rodents have at least four circulating lectin pathway recognition molecules, with differing, but overlapping, carbohydrate specificities; two mannan-binding lectins (MBL-A and MBL-C), collectin-11 (CL-11) and ficolin A (Fcna) [6]. A second murine ficolin, Fcnb, associated with monocyte and macrophage cell surfaces does not activate complement in mice, but may act as a lectin pathway recognition molecule in rats [7]. Humans have a single MBL (the product of *MBL2*; *MBL1* is a pseudogene), CL-11 (collectin kidney 1, CL-K1) and three ficolins, FCN1 (M-ficolin), FCN2 (L-ficolin) and FCN3 (H-ficolin) [5], [8], [9].

These recognition molecules form complexes with three serine proteases, MASP-1, -2 and -3 (MBL-associated serine proteases 1, 2 and 3). The recognition molecules also interact with MAp19 and MAp44 (alias MBL/ficolin-associated protein 1), which are non-enzymatic, truncated alternative splice products of the *MASP2* and *MASP1/3* genes, respectively. Both truncated gene products lack the serine protease domain and may regulate lectin pathway activation by competing for the binding of MASPs to the carbohydrate recognition molecules [4], [10]–[15].

Of the three MASPs, only MASP-2 is required and essential to form the lectin pathway C3 and C5 convertases (C4b2a and C4b2a(C3b)_n_) [6], [10], [16], [17].

Like C1s, activated MASP-2 cleaves C4 and C4b-bound C2, generating C4b2a, the classical and lectin pathway C3 convertase. Neither MASP-1 nor MASP-3 can cleave C4 and therefore cannot compensate for the absence of MASP-2. Thus, formation of the lectin pathway C3 and C5 convertase complexes is impossible in absence of MASP-2 [6]. MASP-1 appears to facilitate lectin pathway activation by either direct cleavage of complex-bound MASP-2 or cleavage of C4b-bound C2, but MASP-1 cannot drive lectin pathway activation in the absence of MASP-2, as MASP-2 is required to initiate the formation of the lectin pathway convertases by the cleavage of C4 [6], [18], [19]. Interestingly, recent work demonstrated that MASP-1 (and possibly MASP-3) play a key role in the maturation and initiation of the alternative activation pathway [20], [21].

Infection studies using mice with targeted deficiencies in one or more complement components have provided evidence for the roles of the classical and alternative pathways in protection against *S. pneumoniae*. C1q deficient mice were found to be more susceptible to infection with *S. pneumoniae* than WT mice, indicating that the classical pathway has a protective role. The alternative pathway was also found to have a protective role against *S. pneumoniae*, but to a lesser extent than the classical pathway. Mice deficient in factor B had a significantly higher level of bacteria in lungs and in blood in comparison to their WT controls [22].

The contribution of the lectin activation pathway towards the defense against *S. pneumoniae* infection had not been assessed so far, mainly due to the lack of appropriate mouse models of total lectin pathway deficiency.

Using MASP-2 deficient mice, completely devoid of the ability to form lectin pathway C3 and C5 convertases, this report demonstrates that lectin pathway activation provides a critical degree of protection against *S. pneumoniae*. We identified mouse ficolin A and CL-11, but not MBL-A or MBL-C, to be the critical pattern recognition molecules that initiate complementactivation via the lectin pathway on the surface of this pathogen.

## Results

### The lectin pathway drives complement deposition on *S. pneumoniae*

**Figure 1:**
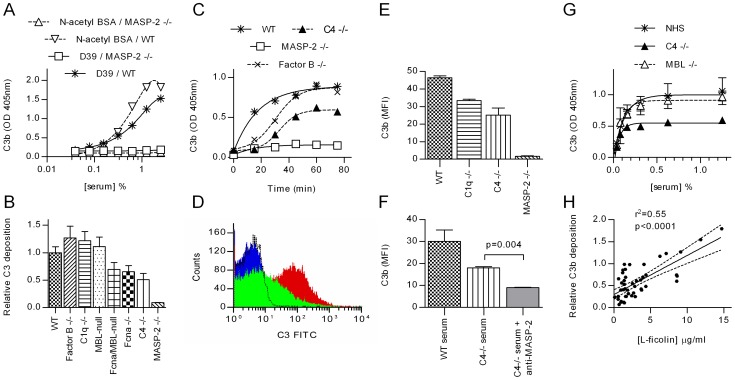
MASP-2 is essential for C3 deposition on *S pneumoniae*. Serial dilutions of sera were incubated in microtiter plates coated with *S. pneumoniae* D39 or N-acetylated BSA (as a control), and C3 deposition determined by ELISA. **A.** Shows the raw data from one experiment with WT and MASP-2 deficient murine serum (means ±SEM). In **B**, the experiment was extended to include murine sera deficient in other complement components. Results are duplicates (±SD) and are normalised to the C3 deposition observed in the WT control. **C.** Time course of C3 activation on *S. pneumoniae*. 1∶40 diluted murine sera were incubated in microtiter plates coated with *S. pneumoniae* for the times indicated then C3 deposition assayed. Results are means of duplicates and are representative of three independent experiments. **D.** FACS analysis of C3 deposition on *S. pneumoniae* opsonised with MASP-2 −/− serum (black line), WT serum (red) and C4 −/− serum (green). The blue trace shows non-opsonised bacteria. **E.** Results from 3 independent FACS analyses of C3 deposition (mean fluorescent intensity ±SEM). **F.** Inhibition of MASP-2 activity with mAb AbD04211 abolished C3b deposition on *S. pneumoniae* opsonised with C4 deficient serum. (means of triplicates ±SEM; p value from Student's t-test). **G.** In human serum, MBL deficiency had no effect on C3 deposition, while C4 deficiency had no significant effect on the EC_50_, but reduces absolute C3b deposition by about 50% (means ±SEM; n = 3 for NHS and MBL −/− serum, 1 for C4 deficient serum). **H.** Correlation between L-ficolin serum concentration and C3b deposition on *S. pneumoniae* immobilised on microtiter plates for 47 samples of NHS (solid line shows Fisher transformation of Pearson's correlation coefficient; dashed lines, 95% CI thereof).

Complement deficient sera were used to determine which components contribute to C3b opsonisation of *S. pneumoniae*. C3b deposition was assayed by ELISA using formalin-fixed bacteria and by FACS analysis using live bacteria. MASP-2 deficiency leads to a total loss of C3b deposition (fig. 1a–e). Using a simple end-point ELISA, C3b deposition appears to be unaffected by factor B deficiency (fig. 1b). However, the conversion of C3 is slower in factor B deficient serum (t_1/2_≈28 min) than in WT serum (t_1/2_≈8 min), indicating that the alternative pathway amplification loop contributes to the C3 turnover (fig. 1c). Serum from MBL-null mice (deficient in both MBL-A and MBL-C) opsonised the bacteria as efficiently as WT serum, whereas ficolin A deficiency resulted in impaired C3b deposition. Ficolin B was not detectable in sera of WT and ficolin A deficient C57BL/6 mice (data not shown). Serum deficient in MBL-A, MBL-C and ficolin A produced similar results to those seen using ficolin A deficient serum, suggesting that the remaining lectin pathway recognition component CL-11 may drive residual lectin pathway activation on *S. pneumoniae* (fig. 1b). In addition to the recent publication by Hansen et al. (2010) [9] which demonstrated MASP-1 binding to CL-11, we have shown that recombinant human CL-11 binds recombinant MASP-2 to form a lectin pathway activation complex under physiological conditions (fig. S1).

Using C4 deficient mouse serum, the absolute amount of C3b deposited on *S. pneumoniae* was approximately half that observed using WT serum (fig. 1b, c, e & f) and the rate of conversion was lower (T_1/2_≈30 min in C4 deficient serum; 8 min in WT serum; fig. 1c). C4 is an integral part of the classical and lectin pathway C3 convertase, C4b2a, suggesting that the residual C3b deposition seen in C4 deficient serum is a result of either alternative pathway activation [23], [24] or the recently reported lectin pathway-specific C4-bypass [6]. Since (i) all experiments reported here were carried out at low serum concentrations (1.25%, fig. 1b; 2.5%, fig. 1c and; 5%, fig. 1e & f) where the alternative activation pathway is dysfunctional, and (ii) the activation of C3 on the surface of *S. pneumoniae* is absent in MASP-2 deficient mice (see fig. 1), we conclude that the C3 deposition on *S. pneumoniae* in C4 deficient serum is mediated by the MASP-2 dependent C4-bypass activation route. Likewise, inhibition of MASP-2 activity with the anti-MASP-2 mAb AbD04211 abolished C3b deposition on *S. pneumoniae* incubated with C4 deficient serum, supporting the hypothesis that the MASP-2 dependent C4-bypass plays a significant role in the opsonisation of *S. pneumoniae* (fig. 1f).

**Figure 2:**
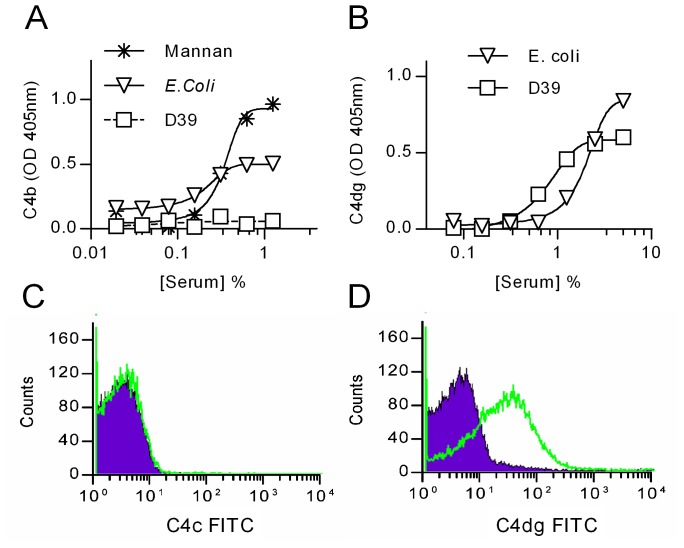
Deposition of C4 breakdown products on *S. pneumoniae*. ELSIA plates were coated with *S. pneumoniae* D39, *E. coli* or mannan (as positive controls) and incubated with NHS for 1 h at 37°C. Bound C4 was detected using an antibody against C4c (which also detects C4b) (**A**) or an antibody against C4dg, the last breakdown product of C4 that remains covalently attached to the activating surface (**B**). **C** and **D**: Detection of C4c (**C**) and C4dg (**D**) on live *S. pneumoniae* D39 opsonised with NHS. Green trace shows anti-C4 antibodies; the purple shading, an isotype control Ab.

Similar results were obtained using human serum from a donor with a complete deficiency of both C4 genes, *C4A* and *C4B*: C4 deficiency halved C3b deposition, although the EC_50_ value was unaffected (≈0.05% for all sera assayed; fig. 1g). Human MBL deficiency had no impact on the deposition of C3b on the surface of *S. pneumoniae* (see fig. 1g). In contrast, analysis of 47 samples of normal human serum (NHS) revealed a significant correlation between serum L-ficolin concentration and the level of C3b deposition *S. pneumoniae* (fig. 2f; p<0.0001; Fisher transformation of Pearson's correlation coefficient). The test group included 8 MBL deficient sera (YO/YO or YO/XA genotypes, see [25]), none of which showed abnormally low C3b deposition, providing further evidence that that human MBL does not contribute to complement activation on *S. pneumoniae*.

### Haemolytically active C4b is undetectable on the surface of *S. pneumoniae* after serum exposure

Antibodies directed against the larger C4 activation fragments, C4b and C4c, failed to detect any C4 deposition on the surface of *S. pneumoniae* exposed to normal human serum (fig. 2a & c). However, when using an antibody directed against C4dg, the haemolytically inactive final degradation product of C4, an abundant deposition of covalently bound C4dg was detected (fig. 2b & d), supporting the hypothesis that *S. pneumoniae* sequester host complement control proteins to accelerate the degradation and inactivation of C4b [26], [27].

### Binding of lectin pathway recognition molecules to *S. pneumoniae*

**Figure 3:**
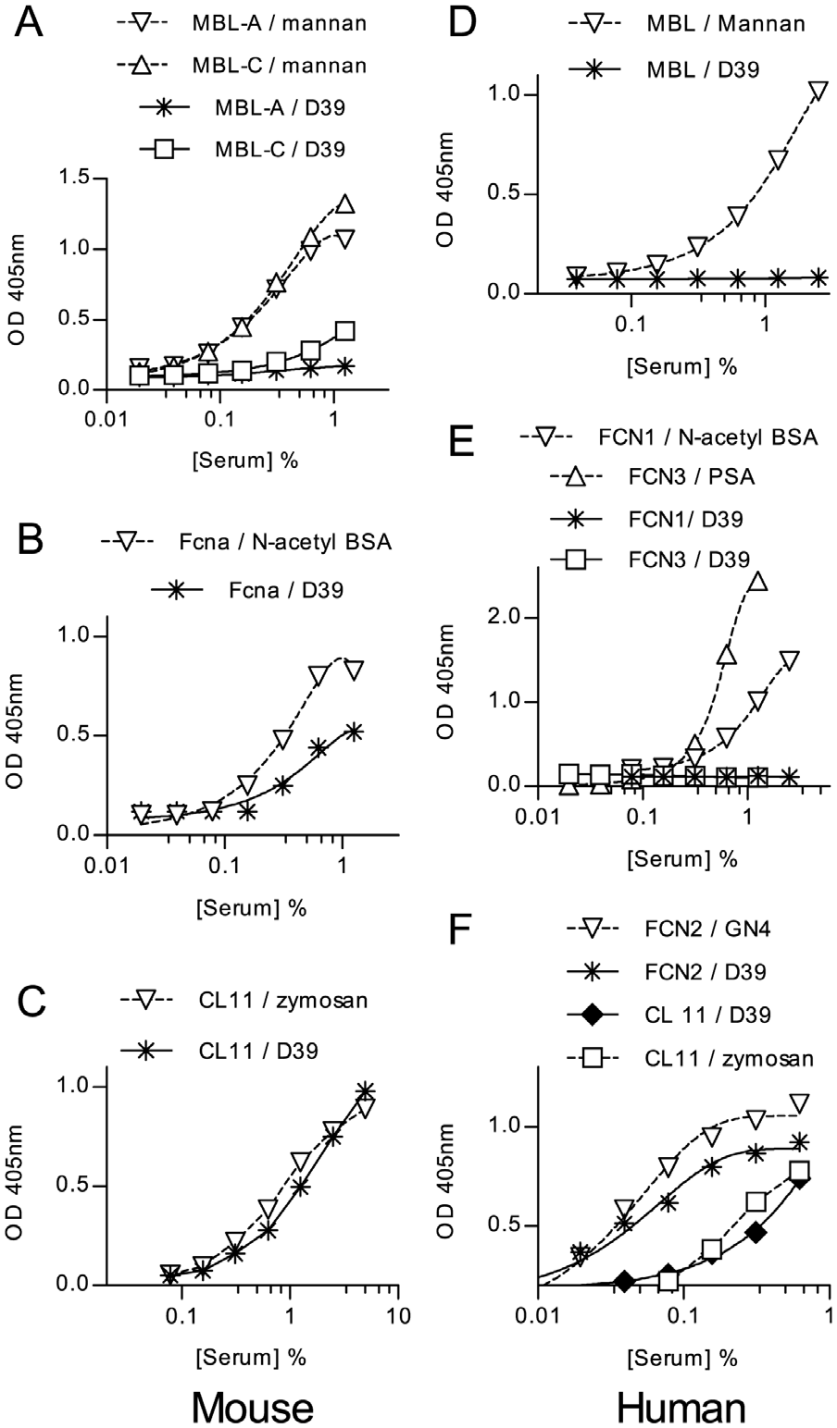
Binding of lectin pathway recognition molecules to *S. pneumoniae*. Microtiter plates coated with formalin-fixed *S. pneumoniae* or control substrates were used to capture lectin pathway recognition complexes from WT mouse serum or NHS. Murine MBL-A and MBL-C (**A**), murine ficolin A (**B**), murine CL-11 (**C**) human MBL (**D**), FCN1 & 3 (**E**) and FCN2 and CL-K1 (**F**) were assayed by ELISA, as described in materials and methods. Only ficolin A, CL-11 and FCN2 (L-ficolin) bound to the bacteria. Results are means of duplicates and are representative of three independent experiments.

A series of solid-phase binding experiments were performed to identify lectin pathway recognition molecules that bind to *S. pneumoniae*. Microtitre plates coated with *S. pneumoniae* D39 and control substrates were used to capture lectin pathway recognition molecules from WT mouse serum and NHS (fig. 3). As anticipated from the C3b deposition assays (fig. 1b & g) and previous reports [28], MBL does not recognize the bacteria, whereas murine Fcna (fig. 3b) and CL-11 (fig. 3c), and human L-ficolin and CL-11 (alias CL-K1) (fig. 3f) do. Direct binding to the bacterial surface was confirmed using recombinant human CL-11 and mouse ficolin A (data not shown).

Nine other strains of *S. pneumoniae* representing three additional serotypes (18, 6B and 3) were tested for binding of human and murine recognition molecules. With human serum the results were remarkably consistent: Of the 5 different lectin pathway specific carbohydrate recognition subcomponents, only L-ficolin and CL11 (alias CL-K1) bound to the bacteria (table S1). Most of the strains showed little or no binding to murine MBL-C and none bound MBL-A. All of the strains tested bound murine CL-11 with similar affinities, and all strains tested bound Ficolin A. MBL-C and Ficolin A binding differed amongst strains of the same serotype, indicating that capsular polysaccharide is unlikely to be the main determinant of recognition by lectin pathway recognition subcomponents.

### MASP-2 is essential for opsonophagocytosis of *S. pneumoniae*

**Figure 4:**
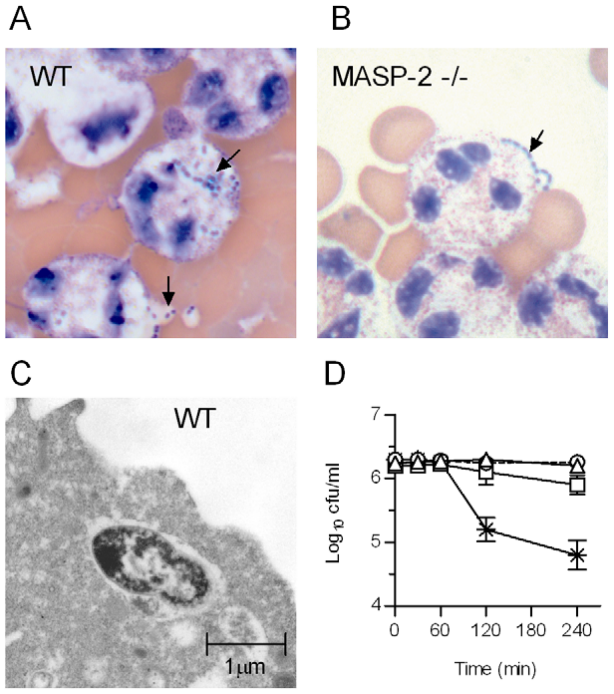
MASP-2 deficiency impairs phagocytosis of *S. pneumoniae* by polymorphonuclear leukocytes. *S. pneumoniae* were pre-incubated with murine serum (20% v/v), mixed with freshly isolated human PMN and incubated for 2 hr before being immobilised on microscope slides and stained with eosin Y and azur II (REASTAIN), as described in materials and methods. Bacteria opsonised with WT serum (**A**) are internalized by PMNs, whereas bacteria opsonised with MASP-2 −/− serum (**B**) are excluded (arrows). **C.** Electron micrograph showing pneumococci opsonised with WT serum inside a PMN. **D.** Samples were removed from the *S. pneumoniae*/PMN mix at the times indicated and viable bacteria determined. WT serum (crosses) facilitated killing by PMN, whereas MASP-2 −/− serum (open squares) is severely compromised in its ability to opsonise *S. pneumoniae*. Controls were run in parallel containing non-opsonised bacteria (triangles) and bacteria opsonised with WT serum, but without PMN (circles). Results are means (±SEM) of triplicates.

The inability of MASP-2 deficient mouse serum to opsonise *S. pneumoniae* with C3b led to defective phagocytosis *in vitro* (fig. 4). *S. pneumoniae* D39 were opsonised with WT or MASP-2 deficient serum and mixed with freshly isolated human peripheral blood polymorphonuclear leukocytes (PMN). Bacteria opsonised with WT serum were internalised (fig. 4a & c), whereas bacteria opsonised with MASP-2 deficient serum were excluded from the PMN, indicative of defective uptake and phagocytosis (fig. 4b). Samples taken from the mixture over a period of 2 hr were plated on blood agar and viable *S. pneumoniae* counted (fig. 4d). Pneumococci mixed with WT serum are efficiently killed by PMN, while those opsonised with MASP-2 deficient serum survive as well as non-opsonised controls. In addition, 20% MASP-2 sufficient (WT) serum from non-immunized mice has no bacteriolytic activity on *S. pneumoniae*. This observation was confirmed using 50% WT mouse and human serum (data not shown).

### Lectin pathway deficient mice are susceptible to *S. pneumoniae* infection

We used MASP-2 and Fcna deficient mice to determine to what extent the lectin pathway contributes to host defense against *S. pneumoniae in vivo*. In contrast to MBL-null [29] and Fcna deficient mice [30] (both of which having lectin pathway activation complexes formed with the remaining recognition molecules in their blood), MASP-2 deficient animals are unable to form the lectin pathway C3 and C5 convertases [6].

**Figure 5:**
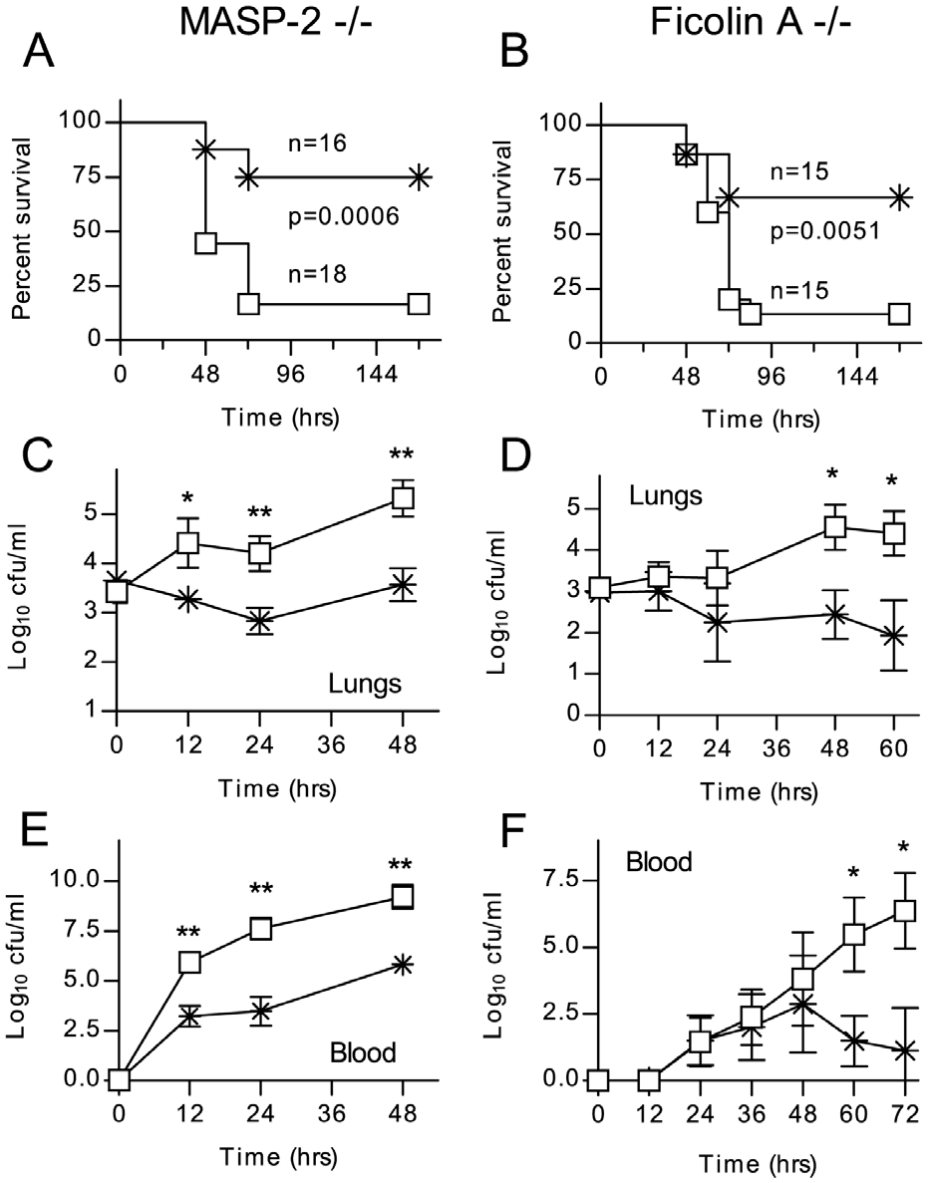
Lectin pathway deficiency significantly increases the severity of *S. pneumoniae* infection. Lectin pathway deficient mice (open squares) and WT littermates (crosses) were infected intranasally with 1×10^6^ cfu *S. pneumoniae* D39. C57/BL6 *^Masp2^* ^−/−^ (**A**) and C57/BL6 *^Fcna^* ^−/−^ (**B**) mice are impaired in their survival of *S. pneumoniae* infection (Mantel-cox log-rank test). **C–F**. Viable *S. pneumoniae* counted in lung homogenates and peripheral blood at the indicated time points after infection. Results in C–E are means (±SEM) of five animals sacrificed at each time point. *p<0.05; **p<0.01 (ANOVA).

Ten-week old C57BL/6*^Masp2^* ^−/−^ mice and WT littermates were infected with *S. pneumoniae* D39 by intranasal inoculation and the course of the infection monitored for one week (fig. 5). 75% of the WT mice survived the infection, compared with only 20% of the MASP-2 deficient mice (p = 0.0006).

During the first 12 hours after infection, the WT mice showed signs of an initial clinical response (hunching), while the MASP-2−/− mice appeared unaffected. In both groups, the first animals reached the endpoint (severe lethargy) after 48 hours, and survival dropped further until 72 hrs after infection. All those animals alive after 72 hr survived (see fig. 5a). In another series of experiments, animals were sacrificed 12, 24 and 48 hr post infection to determine counts of viable *S. pneumoniae* in lung homogenate and blood. In MASP-2 deficient mice, CFUs in the lung (fig. 5c) and blood (fig. 5e) were significantly higher than in WT mice and rose progressively during the first 48 hr, at which point the experiment was stopped due to the high mortality in the MASP-2 deficient group. In WT mice that survived the first 72 hr, bacteria were progressively cleared from the both the lungs and blood. Similar results were obtained with C57BL/6*^Fcna^* ^−/−^ mice; 70% of the WT littermates survived, whereas 80% of the ficolin A deficient mice succumbed to the infection (fig. 5b). Lung infection and bacteraemia are shown in fig. 5d and f.

In contrast, intranasal infection of C57BL/6 *^MBL-null^* (MBL-A and MBL-C double deficient) mice resulted in neither significantly increased mortality nor compromised bacterial clearance mice compared to sex and age matched C57BL/6 WT controls (fig. S2).

Quantitative RT-PCR analysis of cytokine and chemokine expression in lungs from infected animals showed that the onset of the inflammatory response was broadly similar in WT, ficolin A and MASP-2 deficient mice. After 12 hr, however, the pro-inflammatory TNFα response increased more rapidly in the lectin pathway deficient mice, and levels were significantly greater at 24 hr and 48 hr than in the WT mice. In MASP-2 deficient mice, the IL6 response was also elevated. The INFγ response was significantly greater in the ficolin A deficient mice. In the ficolin A and MASP-2 deficient mice, MIP-2 (CLCX2) expression persists at 48 hr, indicating on-going macrophage activation at a time when the response is abating in the WT mice (fig. S3).

**Figure 6:**
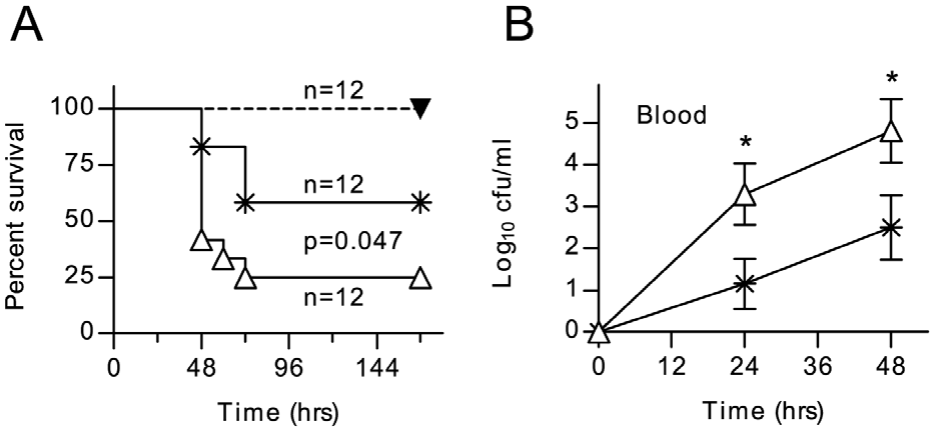
Inhibition of MASP-2 increases the severity of *S. pneumoniae* infection. WT C57/BL6 mice were dosed *i.p.* with 1.0 mg/kg body weight of anti-MASP-2 mAb AbD04211 (open triangles) or an isotype control mAb (crosses) 12 h before being infected with 1×10^6^ cfu *S. pneumoniae* D39. MASP-2 inhibition significantly worsened survival (**A**) and there was a corresponding increase in viable *S. pneumoniae* counted in peripheral blood after infection (**B**). Treatment with 20 mg/kg body weight ceftriaxone 12 hr before infection and every 12 hr thereafter afforded complete protection from *S. pneumoniae* in animals injected with AbD04211 (filled triangles in A). Results in B are means (±SEM) of twelve animals from which blood was taken at each time point. *p<0.05 (ANOVA).

MASP-2 deficiency can be simulated in WT mice using a mAb that specifically inhibits MASP-2. A single *i.p.* dose of 0.6–1.0 mg/kg body weight leads to a loss of ≥90% of lectin pathway activity for up to 7 days [6]. WT mice treated with this mAb prior to infection with *S. pneumoniae* had significantly greater mortality and higher bacteraemia than untreated controls (fig. 6). Antibiotic treatment (Ceftriaxone, 20 mg per kg body weight *i.p.* 12 h before infection and every 12 h thereafter) resulted in complete protection against mortality in both Ab-treated and non-treated groups. These results suggest that increased susceptibility to *S. pneumoniae* in MASP-2 deficient mice is a direct result of the loss of MASP-2 driven lectin pathway activity, rather than an indirect result of MASP-2 deficiency on the development of the animal's immune response.

## Discussion

Complement-dependent opsonophagocytosis is a key feature of the host defense against *S. pneumoniae*. Thirty years ago, using a guinea pig model of pneumococcal bacteraemia, it was shown that the clearance of IgG and IgM opsonized pneumococci from the circulation is entirely dependent upon complement; animals deficient of C4, or depleted of complement using cobra venom factor (CVF), fail to clear the bacteria [31]. More recently, Brown and co-workers [22] used mice with engineered genetic deficiencies to demonstrate the importance of C1q, C4 and C3 in the defense against *S. pneumoniae*. Based on the observation that C1q deficient mice are as susceptible to infection as C4 deficient mice, the authors concluded that activation of the classical pathway is the predominant mechanism for complement-mediated opsonization and phagocytosis of *S. pneumoniae* and that the lectin pathway (which according to the present text book view requires C4 to work) plays a negligible role [22].

Here we analyzed experimental *S. pneumoniae* infection in the first available model of lectin pathway deficiency and reached the conclusion that the lectin pathway promotes innate resistance against pneumococcal infection in the non-immunized host. The results presented here show that MASP-2 deficient mice (which can still activate complement via the classical and alternative pathways [6]) are severely compromised in their ability to survive *S. pneumoniae* infection (fig. 5). Survival times and mortality rates were similar to those reported for C1q and C4 deficient mice, using the same strains of bacteria and mice, and the same dose and route of infection [22].

Neither the classical, nor the alternative activation pathway could compensate for the loss of lectin pathway mediated C3 opsonization of *S. pneumoniae*. In contrast, the absence of C1q had no effect on C3 opsonization of these bacteria *in vitro* (fig. 1) implying that C1q may contribute to bacterial clearance in a process independent of direct C3b or iC3b deposition on the bacterial surface.

Deficiency of factor B, a component of the alternative pathway C3 convertase led to a significantly slower C3 turnover (fig. 1C), This is probably due to the loss of the alternative pathway amplification loop, a positive feedback mechanism that amplifies C3 activation via of all three pathways, which may account for the reported susceptibility of factor B deficient mice to *S. pneumoniae* infection (Brown et al., 2002).

As previously reported by others [26], [32], we were unable to detect any C4b or C4c on the surface of *S. pneumoniae* opsonized with normal human serum (fig. 2). However, the bacterial surface was abundantly decorated with C4dg, the final product of C4 decay. This finding strongly supports the hypothesis that *S. pneumoniae* avoids the accumulation of active C4b by sequestrating complement regulatory proteins from host serum to accelerate the breakdown of C4b, thus preventing the formation of the C3 convertase C4b2a on the pathogen, rather than by preventing C4 binding. Recent work suggests that the pneumococcal virulence factors PspA and PspC are responsible for recruiting factor H and C4-binding protein from host plasma, both of which accelerate the factor I-mediated breakdown of C4b to C4dg [26], [27].

C3 deposition on pneumococci was impaired, but not completely blocked, by C4 deficiency in both mice (fig. 1b–f) and humans (fig. 1g). In the mouse, C4 deficiency led to a significantly slower C3 turnover and in both species the absolute amount of C3 deposited on the bacteria was approximately half of that observed using WT serum. The residual C3 deposition in C4 deficient murine serum could be inhibited using a monoclonal antibody directed against MASP-2 (fig. 1f), indicating that the MASP-2-dependent C4-bypass is active on *S. pneumoniae* [6]. Nevertheless, C4 deficient mice have an increased susceptibility to *S. pneumoniae* infection ([22]; our unpublished data), indicating that the MASP-2-dependent C4-bypass only partially compensates for C4-dependent lectin pathway activation in this setting. The C4-bypass activation of the lectin pathway was shown to play a significant physiological role in ischemia-reperfusion injury; MASP-2 deficient mice are protected from reperfusion injury following myocardial ischemia, whereas C4 deficient mice are not [6], [33].

We hypothesize that the rapid degradation of C4b on the bacterial surface seriously compromises the ability of the classical pathway to form a C3 convertase and thus opsonize *S. pneumoniae* with C3b, while the lectin pathway is still able to opsonize pneumococci with C3b via the C4-bypass, which provides a physiologically relevant degree of compensation for the impaired C4-dependent activation of C3 [6]. The loss of lectin pathway activity caused by MASP-2 deficiency or MASP-2 inhibition would remove a critical degree of C3 opsonization of *S. pneumoniae* in naive mice and hence explain the phenotype of compromised pneumococcal clearance in MASP-2 deficient or MASP-2 depleted mice. The rapid conversion of C4b to C4dg on the pathogen surface appears to be a feature of *S. pneumoniae*. This would explain why the absence of lectin pathway activity renders the host more susceptible to infections with this particular pathogen, whilst no increased predisposition to, or severity of, infection with other major pathogens, e.g. *Pseudomonas aeruginosa* and *Neisseria meningitidis* were observed in MASP-2 deficient mice ([34]; our unpublished data).

In murine serum, it is predominantly ficolin A and CL-11 that bind to *S. pneumoniae* (fig. 3 and table S1). The binding of both lectin pathway recognition molecules to the bacterial surface was confirmed using recombinant human CL-11 and recombinant murine ficolin A. Murine MBL-A does not bind to any of the *S. pneumoniae* strains studied here (covering 4 serotypes), and MBL-C bound weakly or not at all (table S1). It was therefore not surprising that the presence or absence of both MBL-A and MBL-C in C57BL/6 mice had no impact on overall survival in our model of *S. pneumoniae* D39 infection. In contrast, following pneumococcal infection, C57BL/6*^Fcna^* ^−/−^ mice were severely compromised with a significantly higher degree of mortality and higher bacterial loads in blood and lung tissue than WT controls. This phenotype underlines our *in vitro* results and indicates that ficolin A (but not MBL-A or MBL-C) is a key recognition component of the lectin activation pathway in the innate host defense against pneumococcal infection. Interestingly, serum from mice deficient in all lectin pathway recognition components except CL-11 still deposits C3b on the surface of *S. pneumoniae* (fig. 1b). Since mouse CL-11 binds strongly to the surface of *S. pneumoniae* (see fig. 3c) and since we show that CL-11 also forms complexes with the lectin pathway effector enzyme MASP-2 (fig. S1), we conclude that CL-11 acts synergistically with ficolin A as an initiator of lectin pathway activation following binding of specific PAMPs on the pneumococcal surface.

The situation is similar in humans; only L-ficolin and CL-11 recognize *S. pneumoniae* (fig. 3e & f). As previously reported by others [28], we found no binding of MBL to any of the pneumococcal strains (table S1), and there was no indication that MBL deficiency leads to defective C3b deposition on the bacteria (fig. 1g&h). As MBL deficiency is the most common hereditary complement deficiency in humans, affecting as many as 1 in 10 of the population [35], there has been much interest in the possibility of an association between MBL deficiency and infectious disease. In the case of *S. pneumoniae*, the results of association studies have been largely negative or inconclusive, with two of the largest studies finding no association between the risk of community-acquired pneumonia and MBL deficiency [36], [37]. Genetic variations of the L-ficolin gene are more subtle, with no complete functional deficiency in adults reported to date. Furthermore, there is no apparent association between polymorphisms in *FCN2* that lead to low levels of L-ficolin and pneumococcal disease [38], indicating that even low levels of L-ficolin and/or the presence of CL-11 are sufficient to mount a robust immune response against *S. pneumoniae*.

We have previously shown that antibodies to MASP-2 can block lectin pathway driven inflammation and limit tissue loss in ischaemic pathologies [6], suggesting therapeutic utility for anti MASP-2 antibody therapy. Our experiments show that such treatment may increase susceptibility to *S. pneumoniae* infection in naive mice. Pneumococcal vaccination history or immune status may need to be considered prior to initiating anti MASP-2 treatment in patients. Alternatively, since the increased susceptibility was completely reversed by concurrent treatment with ceftriaxone, MASP-2 antagonists should be safe to use with appropriate prophylactic antibiotic treatment.

The results of this study call for a revision of the previously published conclusion by Brown et al. (2002) [22] that the lectin pathway of complement activation is not a major player in the host response to *S. pneumoniae* infection. This conclusion led the same research team to exclude any involvement of the lectin pathway in the clearance of pneumococci in subsequent publications. When studying the impact of human C2 deficiency on C3b opsonization and phagocytosis of *S. pneumoniae* [39], no consideration was given to the fact that C2 deficient individuals are not only deficient of the classical activation pathway, but also of C3 and C5 convertases formed by the lectin pathway. The results presented here, however, strongly suggest that in none-immune sera, it is the loss of lectin pathway functional activity that accounts for the loss of C3b/iC3b opsonization of pneumococci.

Finally, we describe clear evidence of lectin pathway activation in a physiological context in the absence of a discernable contribution by MBL. Thus, the prevailing view that MBL is the predominant initiator of lectin pathway activation may need to be revisited.

## Materials and Methods

### Ethics statement

All animal experiments were authorized by the UK Home Office (Animals Scientific Procedures Act 1986; Home Office project licence 80/2111) and approved the University of Leicester animal welfare committee. Every effort was made to minimize suffering and mice were humanely culled if they became lethargic during infection experiments.

### Materials

Unless otherwise stated, all reagents were obtained from Sigma-Aldrich. PSA, a polysaccharide produced by *Aerococcus viridans* that binds FCN3 was prepared as previously described [40]. AbD04211, a recombinant mAb that potently inhibits mouse MASP-2, has been described previously [6].

### Bacteria

*S. pneumoniae* serotype 2 strain D39 was obtained from the National Collection of Type Cultures, London, United Kingdom (NCTC 7466). Bacteria were identified as pneumococci by Gram staining, catalase testing, alpha-hemolysis on blood agar plates, and determination of optochin sensitivity. Serotypes were confirmed by the Quellung reaction. To obtain pneumococci grown *in vivo*, bacteria were cultured and passaged through mice as described previously [41] and subsequently recovered and stored at −70°C. When required, suspensions were thawed at room temperature and bacteria were harvested by centrifugation before re-suspension in sterile PBS. Nine other clinical isolates of *S. pneumoniae* were kindly provided by Prof. Herminia de Lancastre, Instítuto de Tecnologia Química e Biológica, Oeiras, Portugal [42].

### Mice and murine sera

Mice deficient in MASP-2 and C4 have been described elsewhere [6], [43]. Ficolin A deficient mice were generated by targeting *Fcna* using a conventional replacement vector, as described elsewhere [44]. MBL-null mice were purchased from MMRRC, Bar Harbor, Maine and crossed with *Fcna*−/− mice to produce a strain deficient in all three components. Complement deficient mouse strains were backcrossed with C57/BL6 mice for at least ten generations before use. Blood was collected from these animals and from WT C57/BL6 mice by cardiac puncture, serum prepared, aliquoted and stored at −80°C. C1q deficient and factor B deficient murine plasma was kindly provided by Dr. Marina Botto, Imperial College London.

### Human sera

Human blood was obtained from healthy adult donors who had given written, informed consent, as required by the local ethics committee. L-ficolin concentration was determined as previously described [45]. Genomic DNA was prepared using a kit (Promega) and *MBL2* A/O and X/Y genotypes were determined using fluorescent hybridization probes in a Roche LightCycler [46]. Serum from an individual with a complete deficiency of both C4 genes, *C4A* and *C4B*, has been described previously [47].

### Solid phase binding assays

Nunc Maxisorb microtiter plates were coated with 100 µl of the following reagents: 10 µg/ml mannan (a control for MBL binding), 10 µg/ml zymosan (a control for CL-11 binding), 10 µg/ml N-acetylated BSA (Promega; a control for ficolin A binding), 5 µg/ml of the FCN2-specific mAb GN4, 10 µg/ml PSA, or formalin-fixed *S. pneumoniae* D39 (OD_550 nm_ = 0.6) in coating buffer (15 mM Na_2_CO_3_, 35 mM NaHCO_3_, pH 9.6). Wells were blocked with 250 µl of 1% (w/v) BSA in TBS buffer (10 mM Tris-HCl, 140 mM NaCl, pH 7.4), then washed three times with 250 µl of TBS with 0.05% Tween 20 and 5 mM CaCl_2_ (wash buffer). Serial dilutions of serum in 100 µl of wash buffer were added and the plates incubated for 90 min at room temperature. Plates were washed as above and bound proteins detected using monoclonal rat anti-mouse MBL-A (Hycult), rat anti-mouse MBL-C (Hycult), rabbit anti-mouse ficolin-A, rabbit anti-human M-ficolin, rabbit anti-human L-ficolin, mouse anti-human H-ficolin, mouse anti-human CL-11 or rat anti-mouse CL-11 mAbs. Secondary antibodies were alkaline phosphatase-conjugates and bound antibody was detected using the colorimetric substrate p-nitrophenylphosphate (pNPP).

### Complement assays

To measure C3 and C4 activation, Nunc MaxiSorb microtiter plates were coated with 100 µl of: 10 µg/ml mannan (Promega), or formalin-fixed *S. pneumoniae* D39 (OD_550 nm_ = 0.6) in coating buffer. After overnight incubation, wells were blocked with 0.1% HSA in TBS then washed with wash buffer. Serum samples were diluted in BBS (4 mM barbital, 145 mM NaCl, 2 mM CaCl_2_, 1 mM MgCl_2_, pH 7.4), added to the plates and incubated for 1.5 h at 37°C. The plates were washed again, and bound C3b or C4b was detected using rabbit anti-human C3c (Dako) or rat anti mouse C4 (Hycult) followed by alkaline phosphatase-conjugated goat anti-rabbit IgG or alkaline phosphatase-conjugated rabbit anti rat IgG followed by the colorimetric substrate pNPP.

### FACS analysis

*S. pneumoniae* D39 were washed twice with TBS and re-suspended in BBS to a concentration of 10^6^ cfu/ml. Two hundred µl of the bacterial suspension was mixed with 10 µl of NHS, WT mouse or complement deficient mouse serum and incubated for 1 h at 37°C. After opsonization, the bacteria were washed twice with wash buffer, re-suspended in wash buffer containing FITC conjugated rabbit anti-human C3c (Dako), mouse anti-human C4dg (Quidel) or mouse anti-human C4c (Santa Cruz) and incubated for 1 h on ice. Where non-conjugated primary antibodies were used, the pneumococci were washed twice and incubated for a further hour with FITC conjugated anti-mouse IgG (Dako). After two further washes, the bacteria were fixed using 1% w/v paraformaldehyde, and fluorescence intensity measured by FACS (Becton Dickinson FACS Calibur).

### Phagocytosis assay and bactericidal activity assay of serum complement

Polymorphonuclear leukocytes (PMN) were isolated from fresh human blood by discontinuous density gradient centrifugation using Histopaque-1119 and Histopaque-1077, according to the manufacturer's instruction. Leukocytes were washed twice with Hank's balanced salt solution (HBSS) containing 1.2 mM Ca^2+^ and 1.2 mM Mg^2+^, pH 7.4 (Invitrogen) and re-suspended in HBSS to a concentration of 10^7^ cells/ml.

Killing of pneumococci by PMN was estimated by measuring the decrease in viable bacteria over time. Pneumococci were opsonized by incubation with 20% v/v WT or complement deficient murine serum at 37°C for 30 min. 1×10^6^ PMNs were mixed with 10^5^ pre-opsonized or non-opsonized *S. pneumoniae* D39 in a final volume of 250 µl in HBSS and incubated at 37°C on a rotary mixer. Samples were taken at 0, 30, 60, 120 and 240 min. To determine viable bacteria, samples were serially diluted in HBSS and plated onto blood agar plates.

For histological staining, 25 µl samples of the PMN/pneumococci mix were attached to glass slides by centrifugation at 1500×g for 3 min in a Cytospin 2 (Shanon). The slides were air dried for 15 min and stained using the RESTAIN Quick Diff. Kit (REAGENA). The slides were washed with water, air-dried and then mounted in DPX resin and photographed by bright-field microscopy.

For Transmission Electron Microscopy (TEM), a PMN/pneumococci mix was centrifuged for 5 min at 250× g. Cells were washed twice with 500 µl of 0.1M PBS (pH 7.2) and then fixed by re-suspension into 250 µl of 2.5% glutaraldehyde in 0.1 M PBS (pH 7.2). Fixed PMNs were then examined by TEM.

To assess whether serum complement alone can reduce the number of recoverable *S. pneumoniae* D39 through complement-mediated lysis, bacteria were incubated for 240 min. in 20% and 50% WT human and mouse sera at 37°C on a rotary mixer, samples taken at 0, 30, 60, 120 and 240 min and plated onto blood agar to determine viable bacteria.

### Infection experiments

Ten to twelve week old female MASP-2 and Fcna deficient mice, and their WT littermates were used. Mice were lightly anaesthetized with 2.5% (v/v) fluothane (AstraZeneca) over oxygen (1.5 to 2 litre/min), and 50 µl PBS containing 1×10^6^ cfu of *S. pneumoniae* D39 was then administered into the nostrils of the mice. The inoculum dose was confirmed by viable count after plating on blood agar. For survival experiments, mice were monitored for clinical signs and culled when they became severely lethargic. This time was recorded as the survival time. To determine bacterial tissue counts, groups of mice were deeply anaesthetized at pre-chosen time intervals and blood was collected by cardiac puncture. Immediately afterwards, the mice were culled by cervical dislocation. Lungs were removed separately into 10 ml of sterile PBS, weighed, and then homogenized in a Stomacher-Lab blender (Seward Medical). Viable counts in lung homogenates and blood were determined by serial dilution in sterile PBS and plating onto agar plates supplemented with 5% (v/v) horse blood (Oxoid) and incubated for 18 h at 37°C in anaerobic conditions.

## Supporting Information

Figure S1 MASP-1 and -2 bind to human collectin 11.
(PDF)

Figure S2 MBL deficiency does not increase susceptibility to pneumococcal infection.
(PDF)

Figure S3 Profile of the inflammatory response in the lungs of infected mice.
(PDF)

Table S1 Binding of lectin pathway recognition molecules to different strains of *S. pneumoniae*.
(PDF)

Table S2 Primers used in this study.
(PDF)
