# Supplementary material for: The Lectin Pathway of Complement Activation Is a Critical Component of the Innate Immune Response to Pneumococcal Infection
Source: PLoS Pathog. 2012 Jul 5;8(7):e1002793. doi: 10.1371/journal.ppat.1002793 (PMC3390405; doi:10.1371/journal.ppat.1002793)
Supplement: Figure S1 — MASP-1 and -2 bind to human collectin 11. (PDF) [file ppat.1002793.s001.pdf]

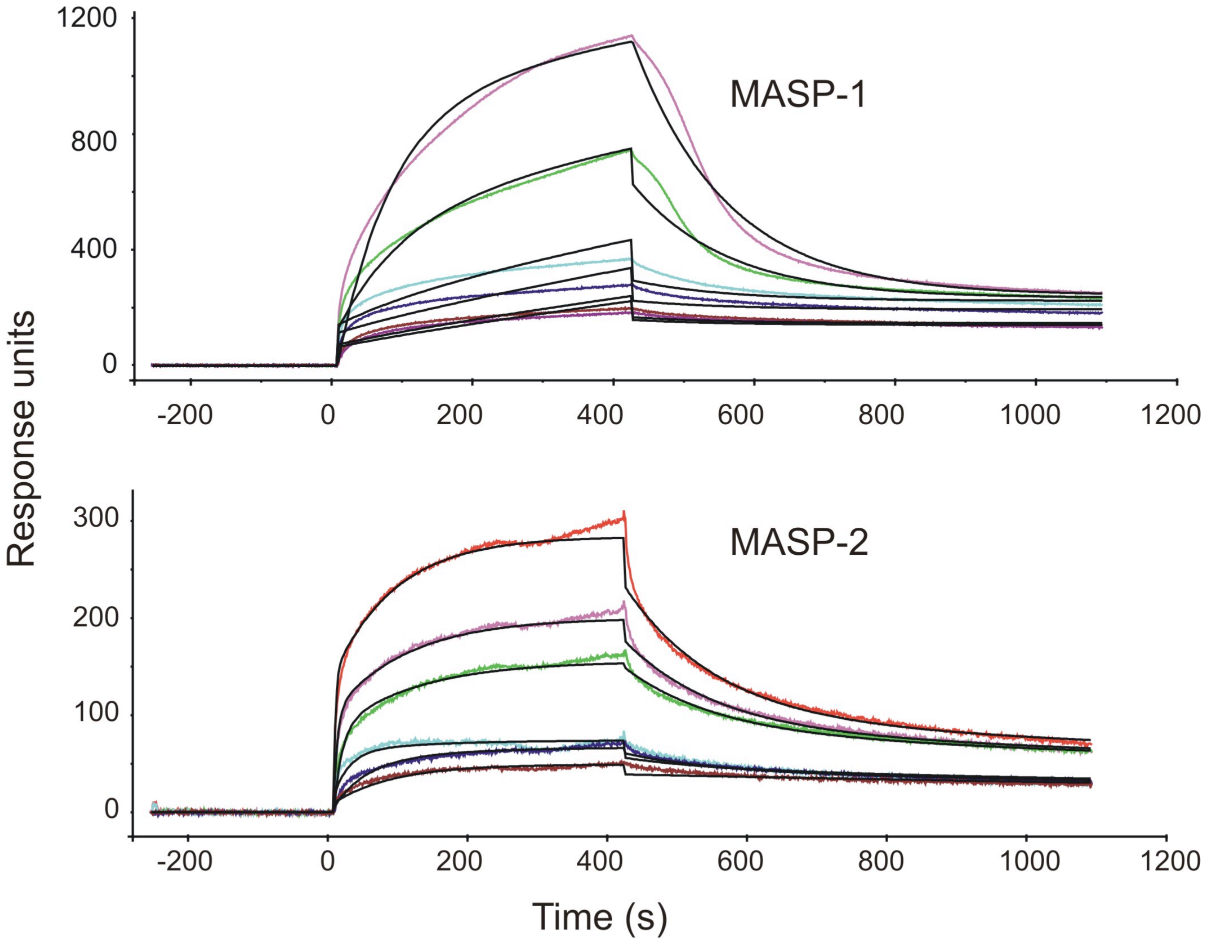

Supplementary figure 1. MASP-1 and -2 bind to human collectin 11.

Binding of MASP-1 and MASP-2 to immobilized collectin 11 was measured by surface plasmon resonance. The cDNA of human collectin 11 was cloned into plasmid PED4 [1] and the protein was purified as described previously for MBL [2]. Collectin 11 (0.025 mg/ml) was immobilized onto the surface of a GLM sensor chip (BioRad; ~8000 response units) at pH 4.5, using amine-coupling chemistry. Binding to MASP-1 and MASP-2 (660, 330, 165, 82.5, 41 and 15 nM) was measured using a ProteOn XPR36 (BioRad) in 10 mM Tris/HCl pH 7.4, containing 140 mM NaCl, 5 mM Ca^{2+} and 0.005% Tween-20 at 25 °C, and at a flow rate of 25 μL/min. Changes in response units caused by differences in sample composition were subtracted from all data. Data are averages from two separate experiments. Black lines show the global fits to the data.

As with MBL and ficolin complexes [3], data fitted to a two-complex, parallel-reaction binding model gave K_D values of 1.1×10^{-8} and 2.2×10^{-7} M for MASP-1 and 1.4×10^{-9} and 5.0×10^{-7} M for MASP-2.

1. Wallis R, Drickamer K. (1999) Molecular determinants of oligomer formation and complement fixation in mannose-binding proteins. J Biol Chem 274: 3580-3589.

2. Kaufman RJ, Davies MV, Wasley LC, Michnick D. (1991) Improved vectors for stable expression of foreign genes in mammalian cells by use of the untranslated leader sequence from EMC virus. Nucleic Acids Res 19: 4485-4490.

3. Girija UV, Mitchell DA, Roscher S, Wallis R (2011) Carbohydrate recognition and complement activation by rat ficolin-B. Eur J Immunol 41: 214-223.
