# Supplementary material for: The Lectin Pathway of Complement Activation Is a Critical Component of the Innate Immune Response to Pneumococcal Infection
Source: PLoS Pathog. 2012 Jul 5;8(7):e1002793. doi: 10.1371/journal.ppat.1002793 (PMC3390405; doi:10.1371/journal.ppat.1002793)
Supplement: Figure S2 — MBL deficiency does not increase susceptibility to pneumococcal infection. (PDF) [file ppat.1002793.s002.pdf]

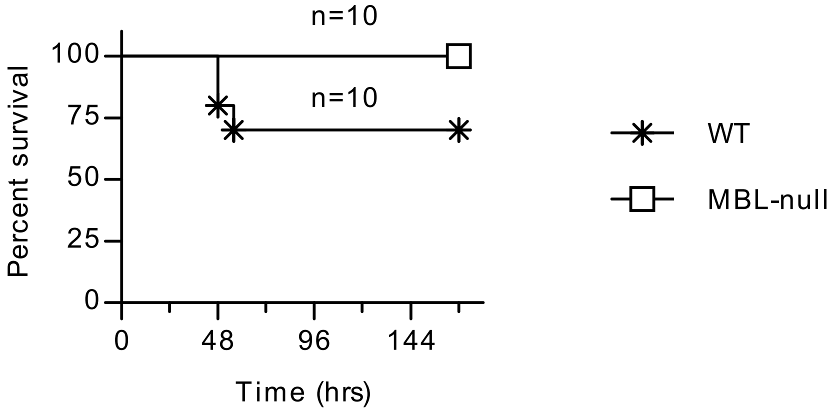

Supplementary figure 2. MBL deficiency does not increase susceptibility to pneumococcal infection.

Ten-week old female MBL-null mice (open squares) and sex and age-matched WT C57BL/6 controls (crosses) were infected intranasally with $1x10^6$ cfu *S. pneumoniae* D39 and the course of the infection monitored for one week. There was no significant difference in mortality between the groups (n=10 per group; $p>0.05$, Mantel-Cox log-rank test).
