# Supplementary material for: The Lectin Pathway of Complement Activation Is a Critical Component of the Innate Immune Response to Pneumococcal Infection
Source: PLoS Pathog. 2012 Jul 5;8(7):e1002793. doi: 10.1371/journal.ppat.1002793 (PMC3390405; doi:10.1371/journal.ppat.1002793)
Supplement: Figure S3 — Profile of the inflammatory response in the lungs of infected mice. (PDF) [file ppat.1002793.s003.pdf]

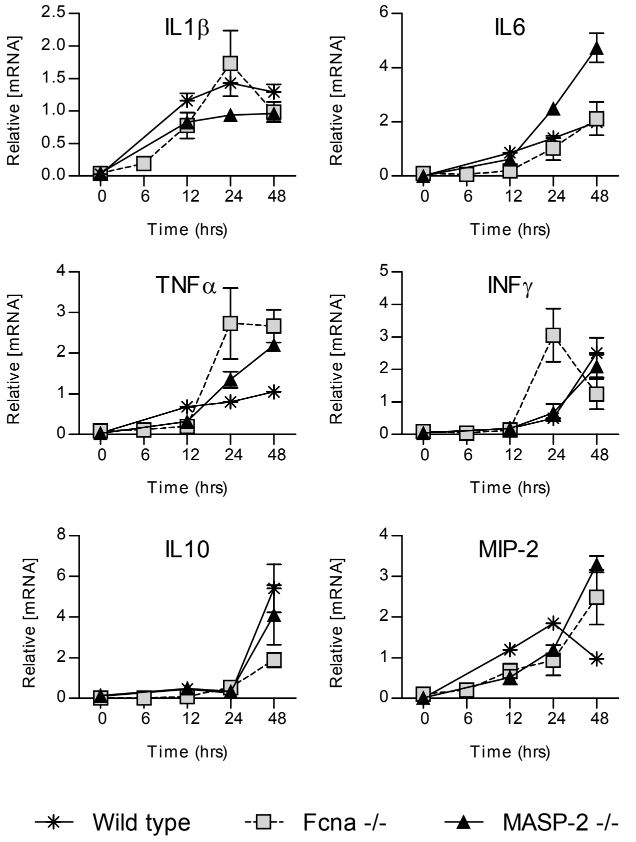

Supplementary figure 3. Profile of the inflammatory response in the lungs of infected mice.

Quantitative RT-PCR was used to follow the inflammatory response in the lung during *S. pneumoniae* infection. Groups of WT, Fcna -/- and MASP-2 -/- mice were infected *i.n.* with $1x10^6$ cfu of *S. pneumoniae*. At pre-determined time points five mice of each genotype were sacrificed and the lungs removed. Total RNA was extracted from 100mg of lung tissue using TRIzol reagent (Invitrogen Life Technologies), according to the manufacturer's instructions. Approximately 10µg of total RNA was digested with RNase-free DNase I (Promega), extracted once with phenol/chloroform/isoamyl alcohol (25/24/1), precipitated, washed with ethanol and then re-dissolved in water. The concentrations of the RNA samples were determined by measuring the absorbance at 260nm. One microgram of each RNA sample was primed with oligo(dT)$_{12\text{-}18}$, and single-stranded cDNAs were synthesized using the SuperScript First-Strand Kit (Invitrogen Life Technologies) according to the supplier's instructions.

cDNA was analyzed by quantitative PCR using a LightCycler (Roche) to follow the incorporation of SYBR Green I into the PCR products in real-time. PCR primer pairs used in this study are listed in table S2. Each 15µl PCR reaction contained 1/100th of the original cDNA synthesis reaction (corresponding to 10ng of total RNA), 0.5µM of each primer, and 7.5µl of QuantiTect SYBR Green Master Mix (Qiagen). Forty-five cycles of amplification were performed; the annealing temperature was reduced from 70 to 58°C during the first 15 cycles and was kept constant at 58°C thereafter. The fluorescence signal was detected at the end of each cycle, and results were analyzed using the Fit Points option in the LDCA software supplied with the machine [1]. Melting curve analysis was used to confirm the specificity of the products [2]. Standard curves were produced for each analysis using serial dilutions of pooled liver cDNA. The concentration of glyceraldehyde-3-phosphate dehydrogenase (GAPDH) cDNA was determined for each sample and used to normalise the results obtained for the experimental samples.

The inflammatory response was broadly normal in all three groups, although expression of the pro-inflammatory cytokine TNFγ increases more rapidly after 12hr in the lectin pathway deficient mice, and is significantly greater at 24hr and 48hr than in the WT mice. IL6 was significantly elevated in the MASP-2 deficient mice, but not ficolin A deficient mice; the reverse was the true for INFγ. In the ficolin A and MASP-2 deficient mice, MIP-2 (CLCX2) expression persisted at 48hr, indicating ongoing macrophage activation at a time when the response is abating in the WT mice. Results are means (±SEM) of five different samples per time point (expect at t=48hr, 2-3 animals per time point in the lectin pathway deficient mice due to high mortality in those groups).

1. Ririe KM, Rasmussen RP, Wittwer CT (1997) Product differentiation by analysis of DNA melting curves during the polymerase chain reaction. Anal Biochem 245: 154-160.
2. Wittwer CT, Herrmann MG, Moss AA, Rasmussen RP (1997) Continuous fluorescence monitoring of rapid cycle DNA amplification. BioTechniques 22: 130-1, 134-8.
