# Supplementary material for: The Lectin Pathway of Complement Activation Is a Critical Component of the Innate Immune Response to Pneumococcal Infection
Source: PLoS Pathog. 2012 Jul 5;8(7):e1002793. doi: 10.1371/journal.ppat.1002793 (PMC3390405; doi:10.1371/journal.ppat.1002793)
Supplement: Table S1 — Binding of lectin pathway recognition molecules to different strains of S. pneumoniae. (PDF) [file ppat.1002793.s004.pdf]

	Serotype 18			Serotype 6			Serotype 3		Serotype 2
	Pt3017	Pt2899	Pt534	Pt586	Pt547	Pt376	Pt815	Pt500	D39
Human									
MBL	ns	ns	ns	ns	ns	ns	ns	ns	ns
L-ficolin	✓✓	✓✓	✓✓	✓✓	✓✓	✓✓	✓✓	✓✓	✓✓
M-ficolin	ns	ns	ns	ns	ns	ns	ns	ns	ns
H-ficolin	ns	ns	ns	ns	ns	ns	ns	ns	ns
CL-11	✓✓	✓✓	✓✓	✓✓	✓✓	✓✓	✓✓	✓✓	✓✓
Mouse									
MBL-A	ns	ns	ns	ns	ns	ns	ns	ns	ns
MBL-C	✓	ns	✓	ns	✓	✓	✓	✓	ns
Ficolin A	✓✓	✓	✓✓✓	✓	✓✓✓	✓	✓	✓	✓✓
CL-11	✓✓	✓✓	✓✓	✓✓	✓✓	✓✓	✓✓	✓✓	✓✓

Supplementary table 1. Binding of lectin pathway recognition molecules to different strains of *S. pneumoniae.*

Nine clinical isolates of *S. pneumoniae* representing three different serotypes (18, 6B and 3), plus the D39 model strain, were tested for binding of human and murine recognition molecules using the solid-phase binding assay described in the main text. With human serum the results were remarkably consistent: Only L-ficolin and CL11 (alias CL-K1) bound to the bacteria. Using murine serum there was some variation in the binding of MBL-C and ficolin A. Two isolates (one type 18, one type 6) had a very high affinity for ficolin A. None of the strains bound MBL-A, while all bound murine CL-11 with similar affinities. (ns= not significant).

MBL-C and ficolin A binding differed amongst strains of the same serotype, indicating that capsular polysaccharide is unlikely to be the main determinant of recognition by the lectin pathway.

Isolates of *S. pneumoniae* were kindly provided by Prof. Herminia de Lancastre, Instítuto de Tecnologia Química e Biológica, Oeiras, Portugal [1].

1. Sa-Leao R, Nunes S, Brito-Avo A, Frazao N, Simoes AS, et al. (2009) Changes in pneumococcal serotypes and antibiotypes carried by vaccinated and unvaccinated day-care centre attendees in portugal, a country with widespread use of the seven-valent pneumococcal conjugate vaccine. Clin Microbiol Infect 15: 1002-1007.
