# Supplementary material for: The Lectin Pathway of Complement Activation Is a Critical Component of the Innate Immune Response to Pneumococcal Infection
Source: PLoS Pathog. 2012 Jul 5;8(7):e1002793. doi: 10.1371/journal.ppat.1002793 (PMC3390405; doi:10.1371/journal.ppat.1002793)
Supplement: Table S2 — Primers used in this study. (PDF) [file ppat.1002793.s005.pdf]

Product (size)	Sequence (5' to 3')
IL1β	CACTCATTGTGGCTGTGGAGA
(247bp)	AGGTGGAGAGCTTTCAGCTCA
TNFα	CCTCACACTCAGATCATCTTCTCA
(237bp)	GTGGGTGAGGAGCACATAG
GAPDH	GTGCTGCCAAGGCTGTG 3
(211bp)	AGACAACCTGGTCCTCAGTGTA
IL6	CAAAGCCAGAGTCCTTCAGA
(95bp)	CACTCCTTCTGTGACTCCA
IL10	CTTGCACTACCAAAGCCACA
(86bp)	TAAGAGCAGGCAGCATAGCA
INFγ	CCTGCGGCCTAGCTCTGA
(81bp)	CAGCCAGAAACAGCCATGAG
MIP-2	ATCCAGAGCTTGAGTGTGAC
(90bp)	AAGGCAAACTTTTTGACCGCC

Table S2. Primers used in this study.
